# Introduction and impact of routine whole genome sequencing in the diagnosis and management of sarcoma

**DOI:** 10.1038/s41416-024-02721-8

**Published:** 2024-07-12

**Authors:** James A. Watkins, Jamie Trotman, John A. Tadross, Jennifer Harrington, Helen Hatcher, Gail Horan, Sarah Prewett, Han H. Wong, Sarah McDonald, Patrick Tarpey, Thomas Roberts, Jing Su, Marc Tischkowitz, Ruth Armstrong, Fernanda Amary, Alona Sosinsky

**Affiliations:** 1 East Genomics Laboratory Hub, Cambridge University Hospitals NHS Foundation Trust, Cambridge, UK; 2 Department of Histopathology, Cambridge University Hospitals NHS Foundation Trust, Cambridge, UK; 3 MRC Metabolic Diseases Unit, Wellcome Trust-Medical Research Council Institute of Metabolic Science, University of Cambridge, Cambridge, UK; 4 Department of Oncology, Cambridge University Hospitals NHS Foundation Trust, Cambridge, UK; 5 Department of Medical Genetics, National Institute for Health Research Cambridge Biomedical Research Centre, University of Cambridge, Cambridge, UK; 6 Department of Histopathology, Royal National Orthopaedic Hospital, Stanmore, UK; 7 Genomics England, London, UK

**Keywords:** Cancer genetics, Diagnostic markers

## Abstract

**Background:**

Sarcomas are diverse neoplasms with highly variable histological appearances in which diagnosis is often challenging and management options for metastatic/unresectable disease limited. Many sarcomas have distinctive molecular alterations, but the range of alterations is large, variable in type and rapidly increasing, meaning that testing by limited panels is unable to capture the broad spectrum of clinically pertinent genomic drivers required. Paired whole genome sequencing (WGS) in contrast allows comprehensive assessment of small variants, copy number and structural variants along with mutational signature analysis and germline testing.

**Methods:**

Introduction of WGS as a diagnostic standard for all eligible patients with known or suspected soft tissue sarcoma over a 2-year period at a soft tissue sarcoma treatment centre.

**Results:**

WGS resulted in a refinement in the diagnosis in 37% of cases, identification of a target for personalised therapy in 33% of cases, and a germline alteration in 4% of cases.

**Conclusion:**

Introduction of WGS poses logistical and training challenges, but offers significant benefits to this group of patients.

## Introduction

Sarcomas comprise a diverse range of neoplasms, which can arise at any anatomical site in the body, show a wide spectrum of histological appearances (even within a single tumour type) and pursue widely differing clinical courses. Moreover, non-sarcomatous malignancies can mimic sarcomas histologically. Accordingly, accurate diagnosis is critically important for patient management but can be challenging for histopathologists, even in specialist settings. Many sarcomas display characteristic genomic abnormalities, which can be disease-defining or even unique to a particular sarcoma, and detection of these can greatly assist in diagnosis. However, the huge (and rapidly growing) number of sarcoma subtypes, coupled with the panoply of non-sarcomatous tumours frequently within the histological differential diagnosis, mean that even the largest targeted panels fail to capture the full genomic landscape necessary for diagnosing and managing these tumours.

In the United Kingdom, Genomics England, a UK government initiative, was established in 2012 for the purposes of setting up whole genome sequencing (WGS) for patients with cancer and rare diseases and standardising high throughput, automated bioinformatic analysis and interpretation. In partnership with the National Health Service (NHS) in England, a large number of tumours, including several sarcomas, underwent WGS as part of the pilot 100,000 Genomes Project. This study revealed the potential value of this assay in the management of sarcoma over current standard of care (SOC) molecular testing, but also highlighted challenges, particularly in bioinformatic analysis and clinical interpretation of variants [1].

Building on the success of the 100,000 genomes project, the NHS Genomic Medicine Service was launched in October 2018 to enable genomic medicine through equitable access to comprehensive genomic testing. Because of the complexity of the genomic landscape in sarcoma, this indication was one of the first cancer types for which WGS was commissioned, but uptake is currently limited.

Our Genomics Laboratory Hub (East Genomics GLH) recently demonstrated the utility of routine WGS in paediatric oncology [2], and we subsequently sought to introduce routine WGS for all sarcomas treated in our regional soft-tissue sarcoma treatment centre and analyse its impact.

## Methods

### Study cohort

Patients undergoing treatment at the Sarcoma Treatment Centre for the East of England at the Department of Oncology at Cambridge University Hospital, UK over a 2-year period (August 2021–August 2023) were offered WGS as part of their routine SOC. Inclusion criteria were: any patient 16 years of age or over with either a known or suspected sarcoma of either bone, soft tissue or visceral organ site. Patients presenting with a suspicious mass underwent a combined procedure to obtain both frozen and fixed tissue to allow up front WGS. In patients with confirmed/suspected diagnoses of sarcoma on conventional histopathology, either a repeat biopsy procedure was performed to obtain frozen tissue or this was taken at the time of a subsequent resection. Exclusion criteria were patients whose disease was not amenable to taking the additional tissue for WGS (either upfront or as a repeat procedure) for reasons of small tumour size and/or accessibility/risk of complications, or who were judged too clinically unwell to undergo a repeat biopsy solely to obtain tissue for WGS. Consent was obtained as per Genomics England protocol (https://www.england.nhs.uk/publication/nhs-genomic-medicine-service-record-of-discussion-form/) and added to the patient record as a record of discussion (RoD).

### Sequencing

DNA from the paired tumour (fresh-frozen tissue) and matched normal (blood) samples were prepared locally using established diagnostic protocols. Prior to submission for WGS, frozen sections were cut and assessed for tumour cell percentage (>30%) and adequacy (<20% necrosis). DNA sequencing was performed centrally at the NHS Genomics Medicine Sequencing Centre in Hinxton, Cambridge, UK. Sequencing library preparation was performed without polymerase chain reaction. Sequencing was performed to a mean coverage of approximately 100× in the tumour and 40× in the paired normal sample.

### Data analysis

Somatic variant calling was performed using a suite of established variant calling algorithms to deliver small variants (single nucleotide variants (SNVs) and indels), copy number aberrations (CNAs) and structural variants (SVs) [3–5]. WGS analysis results were returned as annotated HTML files with high-quality small variants identified by Strelka v2.9.9 triaged into three domains, namely variants in genes indicated for testing in the National Genomic Test Directory for Cancer (Domain 1), other COSMIC Cancer Census genes (Domain 2) or all other protein-coding genes (Domain 3) [6]. Global patterns of mutation were annotated for tumour mutation burden (non-synonymous small variants per Mb of coding sequences) and COSMIC mutational signatures (version 2) [7]. Homologous recombination deficiency (HRD) was assessed using the CHORD algorithm [8]. Germline variant delivery focused on rare (population frequency < 1%) non-synonymous non-benign (according to ClinVar classification) small variants in specific genes pertinent to sarcoma as defined by PanelApp [9]. CNAs and SVs were detected by Manta v1.5 and Canvas v1.39, correspondingly, and their genome-wide profile was depicted linearly via commercial software (BaseSpace Variant Interpreter-Illumina, Inc.) and overall mutational pattern visualised via circos plots.

In addition, a bespoke interpretation filter detailing recurrent and/or diagnostic variants found across the full range of sarcoma entities was manually curated from the literature by the authors and applied to each case to assist in the interpretation of variants, particularly in cases with large numbers of alterations.

The genomic data were interpreted at a weekly Genomic Tumour Advisory Board (GTAB) meeting attended by clinical scientists and molecular pathologists and the pertinent genomic events in each tumour were agreed by expert consensus using accepted annotation methodology [10]. Only oncogenic/likely oncogenic variants were included.

### Clinical review

A summary of the clinically pertinent findings was reviewed at the bi-weekly tertiary Cambridge University Hospital sarcoma oncology MDT meeting with mandatory clinical representation from oncology, radiology, pathology and clinical genetics, as previously described [2]. Genome data were reviewed in the context of the patient’s clinical and histological findings and clinical impact was formally documented as an MDT meeting outcome and via mandatory questionnaire submission to NHSE using pre-defined impact options.

Where WGS-detected variants were used to inform personalised treatment, they were confirmed via orthologous assay (if such an assay was available) prior to clinical action. Similarly, where alternative diagnostic possibilities were raised by the genomic findings, a thorough clinical, radiological and histological review (with additional immunostains and confirmatory orthologous molecular testing if necessary) was conducted and only when these were all considered concordant was the diagnosis changed.

## Results

### Patient cohort characteristics

The service was opened in August 2021, and cases were recruited over a 24-month period. Of patients for whom sampling of fresh tissue was possible, a total of 73 cases were included, six (8%) of which failed to yield sufficient DNA for WGS. Of the six cases that failed to yield sufficient DNA, all six were core biopsies. Of these, two were diagnosed as well-differentiated liposarcomas (one retroperitoneal, one scrotal), one as a ganglioneuroma, one as a neurofibroma (both retroperitoneal), one as fat necrosis (soft tissue of thigh) and one as an extraskeletal myxoid chondrosarcoma (soft tissue of iliac fossa). 67 cases were included in the final study cohort.

The mean age of the patients was 46 (median 47, range 16–81). 18% (12) of patients fell into the category of teenage/young adult (16–25 years of age). The distribution of tissue sampling type was as follows: 48% (32) core biopsies, 36% (24) resections, 13% (9) excisional biopsies and 3% (2) incisional biopsies. The primary site was sampled in 72% (48) of cases, whilst in 38% (19) metastatic disease was sequenced. In 15% (10) cases, the case was biopsied purely to obtain tissue for WGS, whilst in 85% (57) tissue was obtained at the same time as a diagnostic or therapeutic procedure. The primary clinical question motivating WGS was in 32% (21) of cases seeking a therapeutic target, whilst in 69% ()46 the goal was seeking both a therapeutic target and diagnostic clarification. In 37% (25) of cases, neoadjuvant therapy (either chemotherapy or radiotherapy, the latter delivered either at the same primary site as the sampled disease or from a subsequent distant metastasis) had been administered prior to WGS.

The distribution of anatomical sites of tumour sampling was as follows: soft tissue (31.3%) (21), bone 16.4% (11), retroperitoneum 13.4% (9), abdominal viscera 7.5% (5), lung 7.5% (5), skin 6% (4), breast 6% (4), abdominal cavity 4.5% (3), mediastinum 3% (2), lymph node 1.5% (1), meninges 1.5% (1) and sinonasal 1.5% (1).

The distribution of (post-genome) diagnoses was as follows: ‘rare’ tumours driven by gene rearrangement (e.g. GLI-1 rearranged neoplasm) 15.2% (11), UPS 12.1% (8), osteosarcoma 10.6% (7), liposarcoma 7.6% (5), leiomyosarcoma 7.6% (5), synovial sarcoma 6.1% (4), unclassified sarcoma 6.1% (4), non-sarcoma malignancy 6.1% (4), benign neural tumour 4.5% (3), malignant vascular neoplasm 3% (2), myxofibrosarcoma 3% (2), desmoid fibromatosis 3% (2), organ-specific sarcoma (e.g. phyllodes tumour) 3% (2), benign vascular neoplasm 3% (2), solitary fibrous tumour (SFT) 3% (2), rhabdomyosarcoma 3% (2), benign lipomatous tumour 1.5% (1) and malignancy of undetermined lineage 1.5% (1).

### Technical performance metrics of assay pipeline

The time period between the clinical teams having provided all three necessary components to the genomics laboratory (namely, a germline sample, a tumour sample and a completed RoD) and the GTAB meeting (at which actionable variants can be urgently relayed to clinical colleagues) was a mean of 39 days (median = 35 days, range = 20–83 days).

### Overview of genomic somatic variation in cohort

**Fig. 1:**
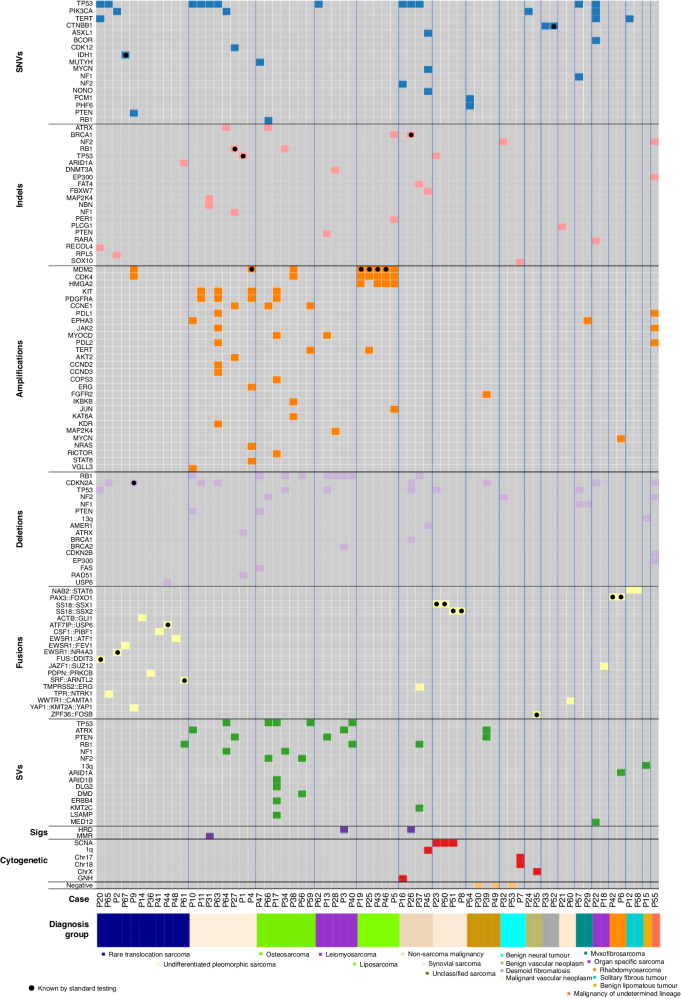
Genomic driver landscape of sarcoma diagnostic cohort.

The genomic driver landscape of the cohort is summarised in Fig. 1.

On average in this cohort, WGS detected medians of 3280 somatically acquired SNVs (range 220–74,784), 299 Indels (insertion/deletions, range 16–175,996) and 132 SVs (range 6–3449) per case. The tumour mutational burden (TMB, non-synonymous variants/Mb) was low with a median of 1.1 (range 0.02–43).

On average, three well-evidenced somatic drivers were identified per case (range 0–11). Of the identified drivers, 25% amplifications, 19% disruptive SVs, 14% SNVs, 11% Indels, 10% fusions, 10% homozygous deletions, 3% heterozygous losses, 4% loss of heterozygosity (LOH), 1% ploidy alterations and 1% segmental copy number alterations (not otherwise specified).

### Impact on diagnosis

**Fig. 2:**
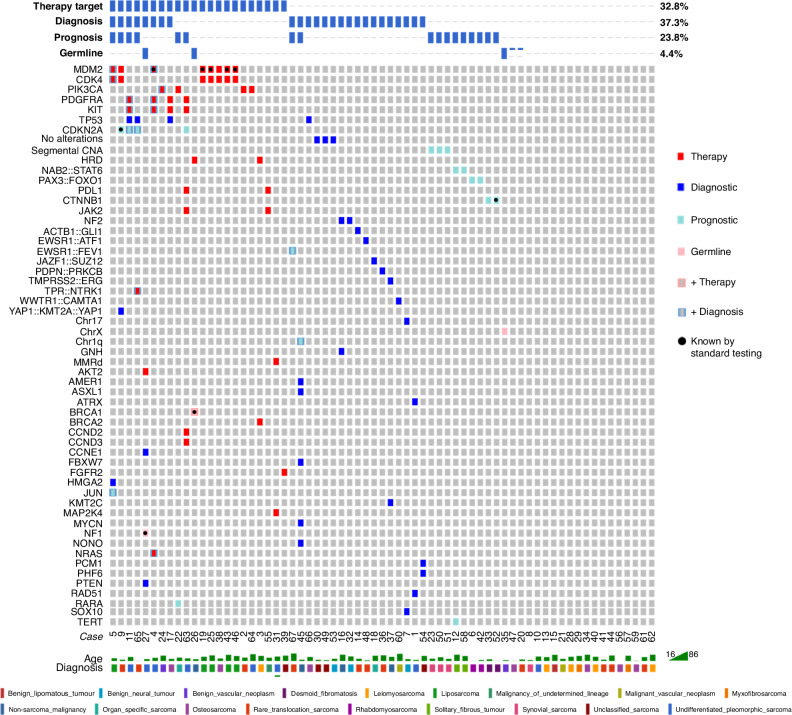
Clinical impact of genomic variants detected by WGS.

In 37.3% of cases (25/67), WGS resulted in an alteration/refinement of the diagnosis above that offered by SOC testing (Fig. 2). Within the cohort, 15% (10/67) of cases had a pre-WGS differential diagnosis of a sarcoma vs a non-sarcomatous malignancy, which was resolved by WGS (six cases confirmed as sarcoma, and four as a non-sarcomatous malignancy) whilst 9% (6/67) of cases had a pre-WGS differential diagnosis of benign vs malignant entities all of of which were again resolved by WGS (all in favour of a benign diagnosis). Details of the cases in which WGS impacted diagnosis are shown in Table 1.

**Table 1:** WGS refined diagnoses

Case	Pre-WGS diagnosis	Selected key diagnostic drivers	Post-WGS integrated diagnosis
1	Recurrent Wilm’s tumour vs undifferentiated sarcoma (radiation-related)	HomDels of *ATRX*, *RAD51* Absence of typical WT drivers [41]	Undifferentiated sarcoma
4	Favour dedifferentiated gastrointestinal stromal tumour (GIST) (DOG1+)	4q Amplification (*KIT/NRAS/PDGFRA*) and *MDM2* amplification Absence of typical GIST drivers [42]	Favour undifferentiated sarcoma
5	Leiomyosarcoma	Amplification of *MDM2/CDK4* and *JUN* [43]	Dedifferentiated liposarcoma
7	Cellular schwannoma vs malignant peripheral nerve sheath tumour (MPNST)	*SOX10* Indel [44] Absence of typical MPNST/eMPNST drivers [45, 46]	Cellular schwannoma
9	Malignant meningioma	*YAP1::KMT2A* fusion [47]	KMT2A-rearranged sarcoma
11	Recurrent metaplastic breast carcinoma vs undifferentiated sarcoma	4q Amplification (*KIT/NRAS/PDGFRA*) [25] + novel *TP53* mutation Absence of *TP53* mutation found in previous primary or other small drivers common in breast carcinoma	Undifferentiated sarcoma
14	Low-grade mesenchymal soft tissue neoplasm, favouring plexiform fibromyxoma	*ACTB::GLI1* fusion [48, 49]	GLI1-altered soft-tissue tumour
16	Poorly differentiated carcinoma of unknown primary vs undifferentiated sarcoma	Truncating *NF2* mutation + haploidisation [12]	Peritoneal mesothelioma
17	High-grade bone sarcoma with suspected BCOR alteration (by IHC)	*TP53* exon 1 truncating mutation [13] + amplifications in 4q/*MYOCD*/*RICTOR*/*COPS3* [50] Wild-type *BCOR* locus	Osteosarcoma
18	Metastatic sex cord-stromal tumour vs endometrial stromal sarcoma	*JAZF1::SUZ12*	Low-grade endometrial stromal sarcoma
24	Hamartomatous vascular malformation	*PIK3CA* mutation [51]	PIK3CA mutated vascular neoplasm
27	MPNST vs undifferentiated pleomorphic sarcoma (UPS) in a patient with NF1	*CCNE1* gain, *PTEN* disruption Absence of variants typical of MPNST [46]	UPS
30	Benign fibrous histiocytoma vs plexiform fibrohistiocytic tumour	No drivers identified [52]	Favour a plexiform fibrohistiocytic tumour
32	MPNST vs cellular schwannoma	Isolated *NF2* disruptive insertion + LOH [53] Absence of typical MPNST-associated SNVs	Cellular schwannoma
36	Cutaneous spindle cell neoplasm of uncertain type	*PDPN::PRKCB* [54]	Benign fibrous histiocytoma (cellular variant)
37	Metastatic sarcomatoid prostate cancer vs radiation-induced sarcoma	*TMPRSS2::ERG* fusion + *KMT2C* disruptive SV [55]	Prostate carcinoma
45	Recurrent Wilm’s vs primary carcinoma/round cell sarcoma	*ASXL1/MYCN/NONO/FBXW7/AMER1*/1q gain [41]	Recurrent Wilm’s tumour
48	Recurrent rhabdomyosarcoma vs melanoma vs NET	*EWSR1::ATF1* [56]	Malignant neuroectodermal gastrointestinal tumour
49	Angiomyxoma vs cellular angiofibroma	Segmental copy number alterations Absence of alterations of *HMGA2* and *RB1* [57, 58]	Unclassified sarcoma
53	Spindle cell tumour infiltrating ganglia vs ganglioneuroma	Absence of any genomic changes	Ganglioneuroma
54	Sarcoma (NOS) favouring dedifferentiated liposarcoma	Alterations in *PHF6*	Unclassified sarcoma
60	Vascular neoplasm, favouring angiosarcoma	*WWTR1::CAMTA1* fusion	Epithelioid haemangioendothelioma
65	UPS	*TPR::NTRK1* fusion [59]	NTRK-rearranged mesenchymal neoplasm
66	Carcinoma of unknown primary	*TP53* intron 1 disruptive SV [13]	Osteosarcoma
67	Sarcoma (NOS)	*EWSR1::FEV1* fusion [11] *IDH1* mutation	EWSR1::FEV1 rearranged soft-tissue tumour

In some cases, WGS allowed a particular entity within the histological differential diagnosis to be favoured or even definitively confirmed. Examples include case 60, a malignant neoplasm with a vascular immunophenotype in which an angiosarcoma was favoured on histological grounds, but was found to have a *WWTR1::CAMTA1* fusion allowing a diagnosis of epithelioid haemangioendothelioma to be made. A further example was case 1, in which it was not possible to distinguish between the recurrence of a Wilm’s tumour and a radiation-induced sarcoma using standard testing; the presence of *ATRX/RAD51B* deletions and the confirmation of the absence of any recurrent Wilm’s tumour associated variants anywhere in the genome allowed a confident diagnosis of an undifferentiated sarcoma.

In other instances, the genomic profile suggested an alternative diagnosis that had not previously been considered, often because a canonical disease-defining variant was detected. Examples include case 67 in which an *EWSR1::FEV1* rearrangement was detected in a soft-tissue neoplasm [11] (in which the rearrangement was not detected by previous external RNA panel testing, possibly because of a breakpoint in the 5’ UTR of *FEV1*, or low expression of the fusion transcript). In other cases, it was the combination of variants that was particularly suggestive of an entity: an example was an abdominal mass with a histological differential diagnosis of sarcomatoid carcinoma of unknown primary or a primary sarcoma (case 16), in which an *NF2* mutation with a haploid reduplicated genome was detected, a pattern particularly characteristic of peritoneal mesothelioma [12]. In case 66, a strongly cytokeratin-positive tumour with widespread visceral and bony metastases was diagnosed as a carcinoma of unknown primary, but following a poor response to treatment WGS was offered and showed a profile in keeping with osteosarcoma, including a *TP53* intron 1 variant particularly characteristic of osteosarcoma [13]. In still other cases (e.g. case 53, a ganglioneuroma), it was the total absence of genomic alterations that allowed a confident distinction from the differential of nerve sheath tumour to be made, an assertion that can only be made with whole genome analysis.

Several variants are known to be of prognostic significance in sarcoma, either within specific subtypes or in sarcoma generally. In this cohort, 23.8% of cases (16/67) had variants detected with proven prognostic significance. Prognostically relevant variants included small variants (e.g. TERT in SFT [14]), losses (e.g*. CDKN2A/B* [15]), amplifications (e.g. *JUN* in liposarcoma [16]), fusion partners (e.g. *PAX3/7::FOXO1* in rhabdomyosarcoma [17]), breakpoint positions in fusions (e.g. *NAB2::STAT6* in SFT [14]) and the extent of segmental (i.e. sub-chromosomal arm) copy number changes (e.g. synovial sarcoma [18]) (Fig. 2).

### Impact on germline cancer susceptibility gene detection

Germline variants of clinical significance were detected in 4.4% of cases (3/67). In two of these cases, cases 26 (*BRCA1*) and 27 (*NF1*), the germline variant had already been previously identified by orthologous testing. The third case was an incidental mosaic XO/Turner syndrome (case 35), which, because of the location of the tumour, impacted on management by introducing greater concern for the preservation of fertility. In addition to these variants, two pathogenic recessive variants were discovered, one in RECQL4 (case 20) and one in MUTYH (case 47), both of which were heterozygous with no evidence of LOH in the tumour.

### Impact on therapeutic target discovery

In 32.8% of cases (22/67), at least one potential target for personalised cancer therapy was revealed by WGS. In patients for whom a target was discovered, as many as 6 targets were identified. Across the cohort, at total of 42 putative therapeutic targets were discovered, of which five (all *MDM2* amplifications) were known by standard testing, representing only 12% of the total targets discoverable by WGS. The landscape of detected therapeutic targets is illustrated in Fig. 2.

The targets identified include *MDM2* [19], *CSF1* [20]*, CDK4/6* [21], *NTRK* [22], *FGFR* [23], *PIK3CA* [24] *KIT/NRAS/PDGFRA* [25], *AKT* [26] and *PDL1* [27, 28]. In addition to these gene-specific targets, several tumours revealed targetable genomic signatures, including HRD predicting response to PARP inhibition [29] and mismatch repair deficiency to immune checkpoint inhibitors [30, 31].

**Fig. 3:**
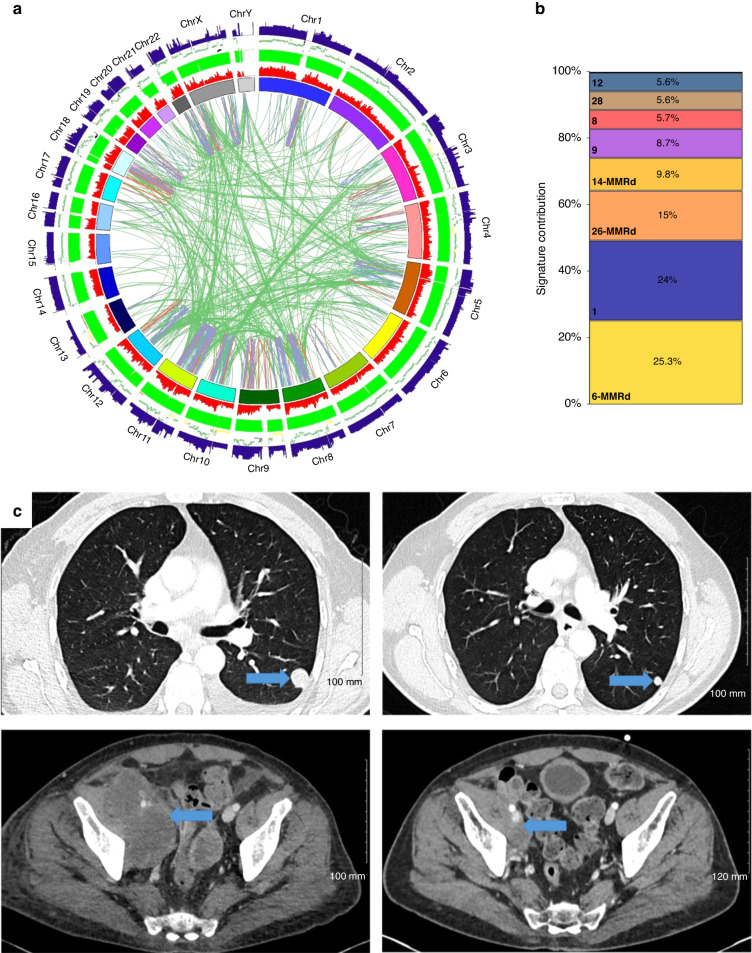
Therapeutic impact of WGS in sarcoma (case 31). **a** Circos plot of WGS showing strong mutational burden of indel variants (green track). **b** Signature breakdown showing strong mismatch repair signature. **c** CT scan images of a 56-year-old male with progressive lung and pelvic metastases from UPS following two previous lines of chemotherapy showing excellent partial response to the immune checkpoint inhibitor pembrolizumab.

An example of WGS results optimising patient management is demonstrated by patient 31, a 56-year-old male with metastatic undifferentiated pleomorphic sarcoma (UPS) with rhabdomyoblastic differentiation. He had already received two lines of standard soft-tissue sarcoma chemotherapy regimen, namely single-agent doxorubicin and gemcitabine with docetaxel, with subsequent disease progression. WGS performed on repeat biopsy revealed a mutational signature profile consistent with mismatch repair deficiency and an elevated TMB (43 non-synonymous variants/Mb). This allowed for a successful application for compassionate access to an immune checkpoint inhibitor, the anti-PD1 antibody pembrolizumab. He was started on pembrolizumab 200 mg every 3 weeks. At the time of writing, his disease is still showing ongoing partial response 8 months later (Fig. 3).

## Discussion

### Routine WGS in the management of sarcoma is feasible and clinically beneficial

We have shown that the introduction of a routine WGS service in the diagnosis and management of sarcoma is feasible and is clinically beneficial, providing insights beyond that offered by non-WGS molecular testing.

We found that WGS altered the diagnosis in 37% of cases analysed and revealed a putative drug target in 33% of cases. We also showed that routine germline WGS had a clinical impact by yielding clinically actionable variants. The detected rate of 4% is slightly less than that reported in other published studies of sarcoma [32, 33], which may be a consequence of the older age demographic in our series compared to these earlier studies which have focused on young adults or the under 50 s.

### Benefits of WGS over conventional panel-based testing

At present, there is no recognised SOC for sarcoma molecular diagnosis internationally, predominantly because the number of targets is large and ever-growing and there is a wide variation in access to and expertise in sequencing technology across centres, but at present most centres use a range of commercially available panels for this purpose. These panels are typically designed to cover the full range of solid and haematological neoplasms, and thus lack a sarcoma focus, and although capable of reliably detecting small DNA variants in common cancer genes, most include only a limited number of fusion and copy number targets with little to no capacity to assess SVs. In contrast, WGS offers several advantages over panel-based testing in the diagnosis of sarcoma.

Firstly, WGS offers comprehensive coverage of all possible targets in the genome. This means that variants in genes only infrequently altered in cancer but which are nonetheless highly useful in diagnosis (e.g. *PHF6/PCM1* in case 9, *NONO* in case 45), can be picked up. Similarly, fusions involving driver genes largely or exclusively restricted to sarcoma (e.g. *KMT2A::YAP1* in case 9) are readily detectable, as are the more sarcoma-specific copy number targets (e.g. *COPS3* in case 17 and *MYOCD* in case 13). The comprehensiveness of a genome is also the gold standard for signature analysis (including emerging signatures relating to copy number and SVs) and the clinical insights it offers [34, 35], with panels offering assessment of only a small number of signatures at lesser accuracy [36]. The access to a full germline genome, annotating both copy number/structural and small variants is also uniquely valuable, as both common but also rare/emerging susceptibility loci can be fully analysed. An important advantage of WGS is the ability to reliably call the absence of any variants, which is highly valuable in distinguishing between benign and malignant entities. As well ascovering all possible target genes, WGS can also detect all possible variant types. In our series, many drivers detected were SVs not readily detected by non-WGS assays, including canonical driver rearrangements not detectable on RNA assays due to either a presumed low-level expression of the chimeric product or possibly relating to a breakpoint in an untranslated region (e.g. the occult *EWSR1::FEV1* fusion in case 67), and disruptive SVs not readily detectable on sub genome targeted panels, including small interstitial deletions (e.g. a 212 bp deletion in RB1 in case 10), non-chimeric disruptive translocations (e.g. *NF1* in case 34) some of which are pathognomonic (e.g. *TP53* intron 1 rearrangement in case 66) and large inversions/duplications (e.g. *ARID1A* in case 6 and *PTEN* in case 27).

The second advantage of WGS is the accuracy and ease of variant calling relative to panel testing. Even if a gene is included on a panel, whether or not a variant is interpreted as pertinent is highly dependent on the local genomic context. The absence of this context when attempting to interpret variants on a targeted panel can lead to errors. For example, an accurate assessment of whether amplification is a genuine driver is highly dependent on an accurate read of overall genomic ploidy, local complexity, completeness of the gene sequence amplified and the focality/co-amplified genes in the amplified area. Similarly, the interpretation of fusions and other SVs as true drivers or just passengers can be influenced by the presence of local chromothripsis, or in contrast, the absence of any other variants in the genome, in which scenario a single SV (even if novel) is highly likely to be the driver event. Finally, determination of the oncogenicity of variants of unknown significance in tumour suppressor genes is aided by having access to full characterisation of the remaining allele, and the presence of a germline for comparison also allows definitive confirmation of somatic status, removing the need to rely on VAF and germline population databases to predict likely germline variants from data.

Although the precise number of variants uniquely detected by WGS would vary somewhat depending on the panel(s) used, given that disruptive SVs alone account for 20% of driver alterations detected in our series, in combination with the other variant types/interpretive issues discussed above, it is likely that greater than 20% of genomic drivers in sarcoma may be missed by a non-WGS approach.

Third are the advantages of the clinical diagnostic laboratory from an organisational perspective. The comprehensiveness of WGS means that only a single assay need be run to assess all relevant variants, which streamlines the laboratory pipeline for highly heterogeneous tumour types such as sarcoma (which on a practical level includes ‘undifferentiated’ tumours) and also reduces the need to train scientists on multiple assays. It also means that there is no requirement to continually alter and then reaccredit assays as additional genes of interest in sarcoma must be added to a panel. Lastly, the full genomic portraits provided by WGS offer greater insights into our understanding of sarcoma going forward, with the potential to uncover hitherto undescribed genomic abnormalities defining novel tumour types and also provide the most complete picture to optimise the ongoing searches for genomic correlates of clinical outcomes [34, 35].

### Challenges to introducing routine WGS for sarcoma

Moving to routine WGS in the management of sarcoma poses challenges. It requires changes in long-standing entrenched practices in radiology, surgery, histopathology and oncology and also adaptations in the rapidly growing field of molecular cancer clinical science. In our experience of introducing this service, some cases who would have been eligible for WGS were not able to receive it, and some of the reasons and proposed solutions are discussed below.

The first challenge is that at present performing WGS of sufficient technical reliability for clinical use requires frozen tissue. This has two main consequences: firstly, in the case of biopsies, additional tissue must be sampled (with an attendant increase in clinical workload and possible risk of complications for the patient), and secondly, that specimens must be handled differently by both the sampling clinicians (be they radiologists or surgeons) and histopathology laboratories in order to preserve the cold chain. An added difficulty that must be overcome in WGS for adult sarcoma (that is not an issue in some other NHSE indications for WGS with pre-existing fully centralised services (e.g. paediatric oncology)) is that biopsies/resections can be performed by any clinical team and at any hospital within a specialist sarcoma team/genomic laboratory hub catchment area, as sarcomas can arise at any age and at any site (e.g. breast, gynaecological and gastrointestinal tracts, head and neck) and are also frequently not suspected to be sarcoma at the time of biopsy or surgery, and many of these teams are both unaware of the need for fresh sampling and have no experience in doing so. This can sometimes be solved by either arranging sampling of a subsequent resection or if possible arranging rebiopsy (as was the case in many of our patients in this series), but this is not always possible (e.g. in the case of a complete excisional biopsy, or where the disease has shown complete regression following chemoradiotherapy, or where re-biopsy presents either too much additional clinical risk or is not acceptable to the patient).

Spreading awareness of the need to consider the sampling of fresh tissue upfront where sarcoma is a possible diagnosis and how to appropriately handle these specimens requires a concerted educational outreach effort. This problem may also in the future be at least partly ameliorated by ongoing studies utilising solutions which stabilise DNA at room temperature may obviate the requirement for cold chain logistics (especially from hospitals remote from the site of the genomic laboratory). However, the establishment of clear standard operating procedures coupled with close collaboration with clinical and laboratory colleagues can overcome this difficulty. Patients undergoing WGS also require matched germline blood samples to be taken, along with a detailed and sometimes time-consuming consent procedure, which are new tasks for clinicians and for which wider training is required if this is not to be a further barrier to patients accessing this service.

The third challenge relates to the accurate and clinically useful analysis of the data. Tumour and germline WGS is still a relatively new assay in the NHS for which experience is limited to non-existent in many centres. Detailed guidance and standardised protocols are yet to be widely implemented for clinical scientists/molecular pathologists in the interpretation of all the data provided by whole genome analysis in cancer. The complexity of cancer genomes requires a structured and adaptable approach to the analysis, and in particular, the highly structurally complex genomes typical of adult sarcomas can pose a heavy burden to interpretation if a pragmatic approach is not adopted. Our analysis approach has proven successful in the application of this technique in sarcoma, and we believe that our approach may be applicable to other centres. We also found that the involvement of histopathology is critical in the accurate analysis of sarcoma genomes. Analysis informed by a clear histological/clinical differential diagnosis is required to ensure that a recognised clinicopathologic tumour entity is diagnosed and genomic variants are not interpreted in isolation leading to confusion and an incorrect diagnosis. This approach also makes analysis easier and quicker as non-diagnostically relevant variants can be discounted more readily. There is a need for increased training in genomics for histopathologists to allow them to offer the necessary input to ensure accurate diagnoses are made, and advances in AI-supported histological diagnosis will also assist in analysis in the future.

The fourth challenge is the requirement to deliver a WGS service in a sufficiently rapid turnaround time to meet the needs of patients who often have complex and/or rapidly progressing diseases and require sufficient diagnostic clarity or identification of personalised targets to permit the commencement of treatment. In most instances in our series, this was possible, but proposed strategies for improvement include the introduction of ‘Genomic Practitioners’ to co-ordinate the procedure (e.g. upfront germline sampling and consent, assistance with transport of specimens to laboratories), use of confocal microscopy for rapid assessment of the adequacy of samples (removing the need for frozen section analysis), and more frequent send-outs to the sequencing centre. Finally, improvements in the genomic laboratory including laboratory automation and more powerful decision support software (utilising both AI technology and an increasingly large library of publicly available clinically annotated genomes) along with the introduction of overnight sequencing technologies will deliver improvements in turnaround time.

A final consideration is that of cost. Accurate comparisons of costs between sequencing approaches are challenging due to wide variations in sequencing technologies, differing funding and organisational approaches across healthcare systems and the variations in the clinical approach to sarcoma diagnosis discussed above, but most analyses performed to date suggest that the costs of WGS are higher than those of basic panel-based testing [37, 38]. However, as noted above, an adequate assessment of these complex tumours would require at least two and possibly three different platforms to be performed, which narrows the cost differential, as does the need to maintain only a single rather than multiple assays in the laboratory. Moreover, some studies in different settings have noted that given WGS must subsequently be performed should panels prove insufficient to resolve the case, an upfront WGS approach is actually more cost-effective in cases known to be genomically challenging [39].

### Conclusions and future directions

For the reasons discussed above, WGS is likely to be the standard molecular investigation in the future and can be implemented in routine clinical practice. However WGS does have some limitations, and additional genome-wide assays can supplement WGS and provide a more complete molecular portrait of sarcomas. These include long-read sequencing (to assess long-range or poorly mapped SVs and also provide insight into the phase of alterations), methylome sequencing (to assess gene silencing as a second hit on tumour suppressor genes and utilise methylation signature diagnostic classifiers) and transcriptomics (to assess the RNA consequences of complex DNA rearrangements). It may also be possible to perform these assays on circulating tumour DNA [40], which may in part ameliorate many of the difficulties inherent in obtaining surgical biopsies. The genomic analysis strategy in sarcoma will continue to progress and offer ever more insights for the treatment of this patient group.

### Supplementary information

Clinical and molecular driver data

## Data Availability

The data supporting the findings of this study are available within the Genomics England Research Environment, a secure cloud workspace. To access genomic and clinical data within this Research Environment, researchers must first apply to become a member of either the Genomics England Research Network (https://www.genomicsengland.co.uk/research/academic) or the Discovery Forum (industry partners https://www.genomicsengland.co.uk/research/research-environment). The process for joining the Research Network is described at https://www.genomicsengland.co.uk/research/academic/join-gecip and consists of the following steps: 1. Your institution will need to sign a participation agreement available at https://files.genomicsengland.co.uk/documents/Genomics-England-GeCIP-Participation-Agreement-v2.0.pdf and email the signed version to gecip-help@genomicsengland.co.uk. 2. Once you have confirmed your institution is registered, you can apply through the online form at https://www.genomicsengland.co.uk/research/academic/join-gecip. Once your Research Portal account is created you will be able to log in and track your application. 3. Your application will be reviewed within 10 working days. 4. Your institution will validate your affiliation. 5. You will complete our online Information Governance training and will be granted access to the Research Environment within 2 h of passing the online training. Data that has been made available to registered users within the Research Environment include: alignments in BAM or CRAM format, annotated variant calls in VCF format, signatures assignment, tumour mutation burden, sequencing quality metrics, a summary of findings that are shared with NHS Genomic Lab Hubs, secondary clinical data. Further details of the types of data available (for example, mortality, hospital episode statistics and treatment data) can be found at https://re-docs.genomicsengland.co.uk/data_overview/.
